# Higher fasting blood glucose worsens knee symptoms in patients with radiographic knee osteoarthritis and comorbid central sensitization: an Iwaki cohort study

**DOI:** 10.1186/s13075-022-02951-2

**Published:** 2022-12-13

**Authors:** Daisuke Chiba, Tetsushi Ohyama, Eiji Sasaki, Makoto Daimon, Shigeyuki Nakaji, Yasuyuki Ishibashi

**Affiliations:** 1 Department of Orthopaedic Surgery, Hirosaki University Graduate School of Medicine, 5 Zaifu-cho, Hirosaki, Aomori, 036-8562 Japan; 2 Department of Endocrinology and Metabolism, Hirosaki University Graduate School of Medicine, 5 Zaifu-cho, Hirosaki, Aomori, 036-8562 Japan; 3 Department of Social Medicine, Hirosaki University Graduate School of Medicine, 5 Zaifu-cho, Hirosaki, Aomori, 036-8562 Japan

**Keywords:** Knee osteoarthritis, Pain, Central sensitization, Hyperglycemia

## Abstract

**Background:**

Although cross-sectional and cohort data suggest that higher serum blood glucose levels in patients with knee osteoarthritis (KOA) are associated with more severe knee symptoms, little is known about the longitudinal relationship between serum blood glucose and knee symptoms, particularly considering central sensitization (CS) comorbidity, which also worsens knee symptoms.

**Methods:**

We evaluated the longitudinal relationship between serum blood glucose and knee symptoms by dividing the cohort of patients with KOA into those with and without CS. We hypothesized that higher serum blood glucose levels would worsen knee symptoms. A total of 297 participants (mean age: 59.6 years; females: 211; average BMI: 23.7 kg/m^2^) were enrolled in this study. At baseline, plain radiographs of the bilateral knee joints were evaluated according to the Kellgren–Lawrence grade (KLG). All participants exhibited at least a KLG ≥ 2 in each knee. At baseline, fasting blood glucose (FBG) and Central Sensitization Inventory-9 (CSI-9) were evaluated; ≥ 10 points on the CSI-9 was defined as CS+. Knee injury and Osteoarthritis Outcome Score (KOOS) was evaluated at baseline and at 1-year follow-up; the change in KOOS (ΔKOOS) was calculated by subtracting the KOOS at baseline from that at the 1-year follow-up. Multiple linear regression analysis was conducted with ΔKOOS as the dependent variable and FBG at baseline as the independent variable, adjusted for age, sex, BMI, and CSI-9 at baseline.

**Results:**

Of the 297 subjects, 48 (16.2 %) were defined as CS+. In the CS − group, there was no association between FBG levels at baseline and ΔKOOS. In contrast, FBG at baseline was negatively associated with ΔKOOS pain (*B* = − 0.448; *p* = 0.003), ADL (*B* = − 0.438; *p* = 0.003), and sports (*B* = − 0.706; *p* = 0.007).

**Conclusions:**

In patients with radiographic KOA and CS, higher blood glucose levels were associated with deteriorated knee symptoms during the 1-year follow-up. Healthcare providers should pay attention to controlling blood glucose, particularly in patients with KOA and concurrent CS, to mitigate their knee symptoms.

**Study design:**

Retrospective cohort study (evidence level: III).

**Supplementary Information:**

The online version contains supplementary material available at 10.1186/s13075-022-02951-2.

## Introduction

Knee osteoarthritis (KOA) is a common form of arthritis that causes chronic knee symptoms such as pain and restricts range of motion, which negatively impacts the patients’ activities of daily living (ADL) [1, 2]. Knee symptoms derived from KOA are a global social burden; therefore, controlling these symptoms contributes to improving individual health status as well as reducing the societal impact [3–6]. Although there are various mechanisms underlying knee pain in patients with KOA, hyperglycemic conditions in diabetes mellitus (DM) have been reported to exert cellular and molecular effects on the nociceptive pathway, thereby intensifying pain [7–9]. Interestingly, a more recent cross-sectional cohort study reported that patients with KOA and concomitant DM experience worse knee pain compared to those without DM, which supports the previous cellular or molecular mechanisms regarding nociceptive pathways [10]. However, the longitudinal relationship between hyperglycemic conditions and worsening of knee pain in patients with KOA remains unclear due to a lack of evidence.

Central sensitization (CS) is another important factor that worsens the symptoms of patients with chronic musculoskeletal disorders. Amplified nociceptive inputs from osteoarthritic joints indicate complex alterations in the central nervous system. Accordingly, nociceptive neurons at various levels of the neuraxis develop a state of hyperexcitability with joint input, consisting of enhanced responses to mechanical stimulation of the joint and lowering of the excitation threshold of spinal cord neurons. The neurons begin to exhibit increased responses to mechanical stimuli in the regions adjacent to or remote from the knee joint. Finally, these changes amplify nociceptive processing [9]. Chronic knee pain derived from KOA consistently shows signs of CS that promotes further development of the patients’ knee symptoms by suppression of the descending pathway in the dorsal root ganglion of the spinal cord [8, 9, 11, 12]. However, little information is available on further translational research regarding the mechanism by which higher blood glucose synergically worsens knee symptoms in patients with KOA based on comorbid CS. Therefore, using a Japanese cohort, this study aimed to evaluate the longitudinal relationship between fasting blood glucose (FBG) at baseline (BL) and the change in knee symptoms based on CS comorbidity. Our hypothesis was that a higher FBG level would be more likely to deteriorate knee symptoms in patients with KOA and comorbid CS.

## Materials and methods

**Fig. 1:**
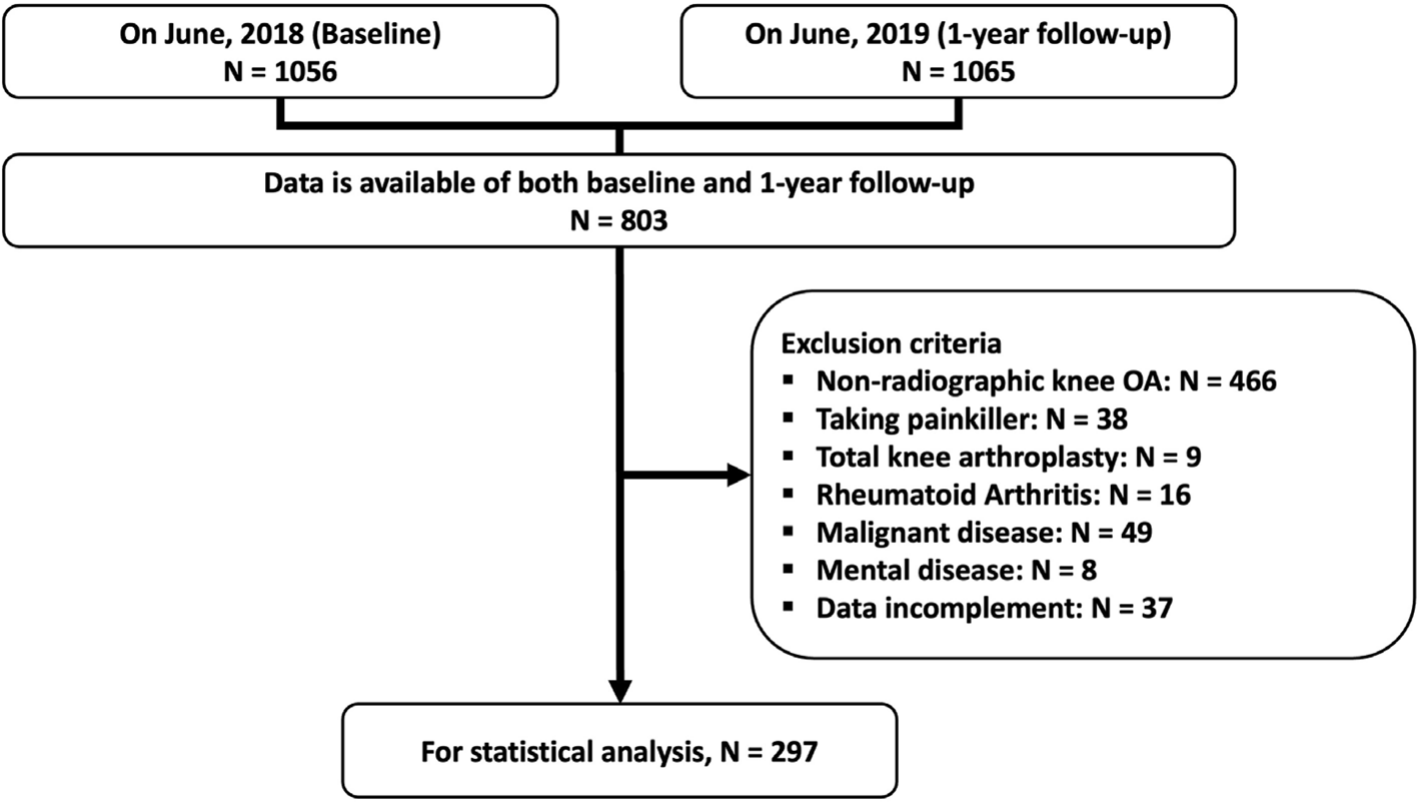
Flowchart of participant recruitment

A total of 1056 volunteers of approximately 12,000 eligible individuals who resided in the Iwaki area of Hirosaki city participated in the Iwaki Health Promotion Project in June 2018 (BL). Of the 1065 participants, 803 were followed up in June 2019 (1-year follow-up [1YFU]); therefore, both BL and 1YFU data were obtained for those participants (Fig. 1). Plain radiographs of both knees were evaluated according to the Kellgren–Lawrence grade (KLG) [13]. A KLG ≥ 2 was defined as definitive radiographic KOA. All subjects exhibited at least a KLG ≥ 2 in each knee. Patients who received oral analgesics and underwent total knee arthroplasty were excluded from this study. Additionally, we excluded patients with a history of rheumatoid arthritis, malignant disease, or mental disease. Finally, 297 participants were included in the current analysis. The ethics committee of the Hirosaki University Graduate School of Medicine approved this study, and all subjects provided written informed consent before participation.

### Evaluation of knee symptoms and central sensitization

All subjects completed the Knee Injury and Osteoarthritis Outcome Score (KOOS) to evaluate their knee symptoms. KOOS included five subscales: pain, symptoms, ADL, sports and recreation (sports), and knee-related quality of life (QOL). The KOOS is a 42-item, knee-specific, self-administered instrument. All items were scored from 0 to 4 and then summed. Raw scores were then transformed into a 0–100 scale, in which 100 represented the best result and 0 represented the worst. A separate score was calculated for each of the five subscales. The KOOS score has been validated as a sufficiently reliable and responsive tool for the assessment of pain, stiffness, and other symptoms including ADL, function for sports and recreation, and QOL associated with various types of knee disorders [14, 15]. Based on the KOOS scales at both BL and 1-year follow-up, we evaluated the change in KOOS (ΔKOOS) by subtracting the KOOS at BL from that at the 1-year follow-up ([ΔKOOS] = [KOOS at 1YFU] – [KOOS at BL]).

All subjects also answered the Japanese version of the short-form Central Sensitization Inventory (CSI-9) questionnaire [16, 17]. The CSI-9 contains nine items related to current health symptoms, and each item is measured on a 5-point Likert-type temporal scale: none (0), rarely (1), sometimes (2), often (3), and always (4), which enables clinicians to determine the occurrence of generalized hypersensitivity related to CS [16, 17]. A cumulative score ranging from 0 to 36 is obtained, and a score of 36 points indicates the worst condition. Based on a previous Japanese cohort study of musculoskeletal disorders, 0–9 points on the CSI-9 were considered non-CS [17]. Therefore, we defined subjects with ≥ 10 points on the CSI-9 as having CS (CS+).

### Evaluation of fasting blood glucose level

At BL, fasting blood samples of all subjects were collected early in the morning before breakfast. The blood samples were centrifuged and serum was obtained for later analysis. The serum samples were stored at − 80 °C. Fasting blood glucose (FBG; mg/dL) was measured using the enzymatic method with glucose oxidase. (LSI Medience Corporation; Tokyo, Japan)

### Statistical analysis

Statistical analysis was performed using SPSS ver. 24.0 (SPSS Inc., Chicago, IL, USA). The distribution of all continuous values was evaluated using the Shapiro–Wilk test. Thereafter, we conducted a non-paired *t*-test or Mann–Whitney *U* test to compare continuous parameters between patients with and without CS. A chi-square test was conducted to compare categorical variables. To clarify the longitudinal relationship between FBG at BL and ΔKOOS, multiple linear regression analysis was conducted with ΔKOOS as the dependent variable and FBG at BL as the independent variable, adjusted for age, sex, BMI, and CSI-9 at BL. Statistical significance was set at *P* <0.05.

## Results

Of the 297 subjects, 48 (16.2%) were classified as CS+. Regarding the demographic data at BL, those with CS were significantly younger and exhibited lower KOOS ADL and sports subscales compared to those without CS (Tables 1 and 2). On the other hand, regarding the longitudinal change in KOOS score, the ΔKOOS showed no difference between those with and without CS (Table 2). The baseline demographic parameters and ΔKOOS between excluded 759 non-OA participants and the current subjects were provided in the Supplemental Table 1. In summary, the current subjects were older, higher number of female subjects, higher BMI, higher FBG, higher CSI-9, and lower baseline KOOS than those without knee OA. Regarding ΔKOOS, there is no significant difference between two groups.

**Table 1:** Demographic characteristics of participants

	CS – (*N* = 249)	CS + (*N* = 48)	*P*-value
Age, years	60.5 ± 12.4	55.2 ± 14.2	0.027^*^
Sex (M/F)	75/174	11/37	0.314
BMI, kg/m^2^	23.6 ± 3.6	24.5 ± 4.4	0.142
KLG	2.2 ± 0.4	2.2 ± 0.4	0.925
FBG, mg/dL	97.1 ± 13.1	95.7 ± 15.4	0.202
CSI-9	3.7 ± 2.8	13.9 ± 4.2	< 0.001^*^

The values are presented by mean ± SD. Statistical analysis: chi-square test and Mann–Whitney *U* test (*P ≤ 0.05); *CS* central sensitization (defined as ≥ 10 points in the CS inventory-9)

**Table 2:** Course of knee symptoms evaluated by knee injury and osteoarthritis outcome score

	KOOS	CS – (*N* = 249)	CS + (*N* = 48)	*P*-value
Baseline	Symptom	86.7 ± 15.7	84.2 ± 15.6	0.087
	Pain	88.5 ± 15.5	85.3 ± 17.2	0.134
	QOL	75.9 ± 24.2	70.3 ± 25.6	0.089
	ADL	93.9 ± 10.6	90.3 ± 14.3	0.043^*^
	Sports	84.1 ± 22.8	77.2 ± 26.1	0.049^*^
1-year	Symptom	83.7 ± 17.1	83.0 ± 14.6	0.343
	Pain	87.3 ± 16.0	85.5 ± 17.4	0.374
	QOL	73.3 ± 25.1	71.5 ± 25.4	0.551
	ADL	93.3 ± 12.3	90.2 ± 14.7	0.180
	Sports	81.9 ± 24.0	77.8 ± 30.7	0.840
Follow-up	ΔSymptom	− 3.0 ± 13.1	− 1.2 ± 15.3	0.147
	ΔPain	− 1.2 ± 12.1	0.2 ± 14.3	0.323
	ΔQOL	− 2.6 ± 19.0	1.2 ± 15.5	0.277
	ΔADL	− 0.6 ± 10.2	− 0.1 ± 13.8	0.249
	ΔSports	− 2.3 ± 17.7	0.6 ± 22.9	0.418

The values are presented by mean ± SD. Statistical analysis: chi-square test and Mann–Whitney *U* test. (*P ≤ 0.05); *CS* central sensitization (defined as ≥ 10 points in the CS inventory-9)

**Fig. 2:**
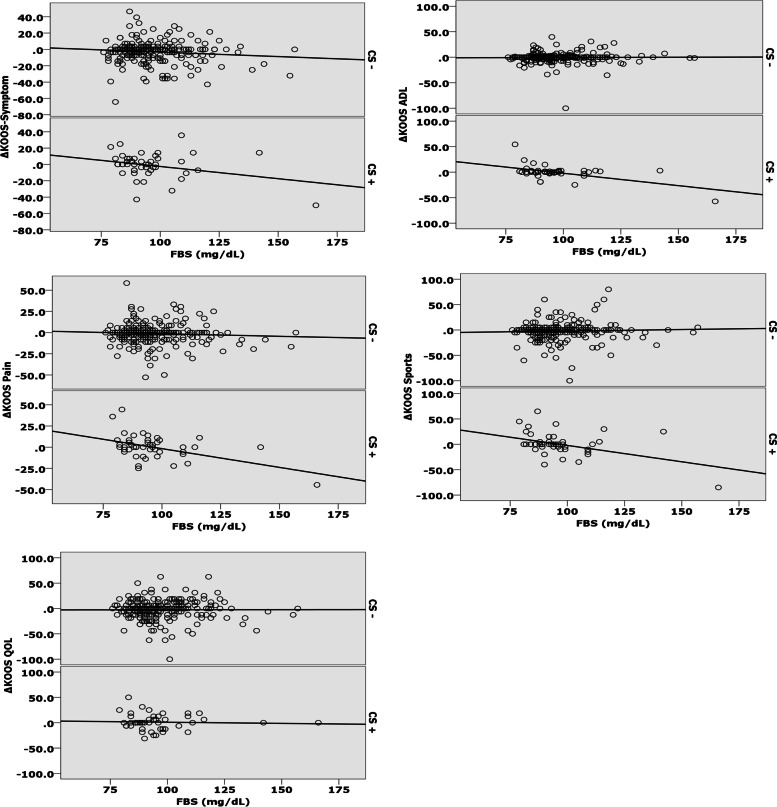
Scattergram showing fasting blood glucose at baseline and ΔKOOS based on prevalence of central sensitization

In the non-CS group, there was no association between FBG levels at BL and ΔKOOS. In contrast, in the CS group, FBG levels at BL were negatively associated with ΔKOOS-Pain, ADLs, and sports (Fig. 2, Tables 3 and 4). For instance, according to the adjusted linear regression model of ΔKOOS-Pain, those having 150 mg/dL-FBG at BL showed deterioration by approximately 22.4 points on the KOOS Pain scale at the 1-year follow-up, compared with those having 100 mg/dL-FBG at BL. ([Adjusted *B*: –0.448] × [Difference in FBG: 150–100] = 18.5; Table 4).

**Table 3:** Crude and adjusted relationship between fasting blood glucose at baseline and ΔKOOS in non-CS group

ΔKOOS	Model	*B*	*P*-value	*95% CI*		
Symptom	Crude	− 0.109	0.085	− 0.233	–	0.015
	Adjusted	− 0.082	0.245	− 0.220	–	0.056
Pain	Crude	− 0.060	0.306	− 0.176	–	0.055
	Adjusted	− 0.069	0.289	− 0.197	–	0.059
QOL	Crude	0.004	0.968	− 0.177	–	0.185
	Adjusted	0.046	0.648	− 0.153	–	0.245
ADL	Crude	0.010	0.836	− 0.087	–	0.108
	Adjusted	0.020	0.720	− 0.090	–	0.129
Sports	Crude	0.059	0.495	− 0.110	–	0.227
	Adjusted	0.070	0.462	− 0.118	–	0.258

Statistical analysis: Linear regression analysis; *B* regression coefficient, *95% CI* 95% confidence interval, *CS* central sensitization (defined as ≥ 10 points in the CS inventory-9)

**Table 4:** Crude and adjusted relationship between fasting blood glucose at baseline and ΔKOOS in central-sensitization group

ΔKOOS	Model	*B*	*P*-value	95% CI		
Symptom	Crude	− 0.299	0.038^*^	− 0.580	−	− 0.017
	Adjusted	− 0.278	0.122	− 0.632	−	0.077
Pain	Crude	− 0.442	0.001^*^	− 0.684	−	− 0.200
	Adjusted	− 0.448	0.003^*^	− 0.737	−	− 0.158
QOL	Crude	− 0.047	0.755	− 0.345	−	0.252
	Adjusted	− 0.077	0.665	− 0.430	−	0.277
ADL	Crude	− 0.486	< 0.001^*^	− 0.710	−	− 0.262
	Adjusted	− 0.438	0.003^*^	− 0.713	−	− 0.163
Sports	Crude	− 0.646	0.002^*^	− 1.044	−	− 0.248
	Adjusted	− 0.706	0.007^*^	− 1.210	−	− 0.202

Statistical analysis: Linear regression analysis; *B* regression coefficient; *95% CI* 95% confidence interval (*P ≤ 0.05)

## Discussion

Based on a Japanese cohort with radiographic KOA, the current study elucidated the relationship between the FBG at BL and longitudinal change in knee symptoms during a 1-year follow-up. The primary finding of this study was that higher FBG levels worsened KOOS pain, ADL, and sports in patients with radiographic KOA and comorbid CS. In contrast, FBG levels were not longitudinally associated with knee symptoms in patients with radiographic KOA without CS. For patients with radiographic KOA, CS comorbidity was negatively associated with the KOOS scores at BL. However, CS did not affect longitudinal changes in KOOS. Therefore, CS comorbidity worsened knee symptoms in patients with radiographic KOA only when their FBG was elevated.

In previous cross-sectional data, patients with osteoarthritis and comorbid DM reported worse knee pain and greater physical and mental issues than those without DM [10]. The current cohort study consistently revealed that the higher FBG at BL longitudinally worsened KOOS pain, ADL, and sports subscales in patients with radiographic KOA with concurrent CS. Regarding the rationale by which DM enhances pain intensity, patients with DM are likely to have more severe synovitis and higher concentrations of interleukin (IL)-6 in the synovial fluid than those without DM, indicating that hyperglycemic conditions enhance the release of cytokines from chondrocytes [9, 18]. The infrapatellar fat pad in osteoarthritic joints is the source of IL-6 and sIL-6 receptors [19]. IL-6 is a major cytokine that induces long-lasting sensitization of joint nociceptors to mechanical stimuli and persistent mechanical hypersensitivity [20]. IL-6 primes nociceptors and prolongs and enhances the sensitizing effect of prostaglandin E_2_ [21]. In addition, DM can strongly influence cellular metabolism and degrade mitochondrial function [22]. Accordingly, the production of reactive oxygen species and their intracellular formation leads to leakage of methylglyoxal (MGO), which results in the formation of advanced glycation end products (AGEs) [23]. MGO and AGEs enhance the excitability of dorsal root ganglion (DRG) neurons and firing of nociceptive neurons by acting on the voltage-gated sodium channel Na_v_ 1.8 [24], facilitating neurosecretion of calcitonin gene-related peptide, and increasing cyclooxygenase-2 expression. Finally, DM evokes thermal and mechanical hyperalgesia [24], and this rationale supports the concept that hyperglycemia itself can be a source of pain.

CS induces hyperexcitability of nociceptive neurons at various levels of the neuraxis and amplifies nociceptive processing. During the course of joint inflammation arising from osteoarthritis, nociceptive spinal cord neurons connected to the knee joint input develop a state of hyperexcitability and lower the excitation threshold against the originally high-threshold spinal cord neurons [9]. Thereafter, nociceptive neurons begin to exhibit increased responses to the mechanical stimuli applied to the osteoarthritic knee joint [9, 25]. Based on the previous rationales, the comorbidity of CS with KOA is expected to independently amplify knee symptoms. However, contrary to the expectation of the pain-modifying effect derived from CS, the current data demonstrated that the comorbidity of CS with radiographic KOA was not associated with the longitudinal worsening of KOOS, whereas this comorbidity was significantly associated with the cross-sectional decrease in KOOS at BL. Most nociceptive sensory neurons are polymodal and very complex with respect to the nociceptive pathway in KOA [26, 27]. Various mediators have been identified and are believed to be involved in the pathological process of KOA. Nevertheless, a limited number have been tested regarding whether they directly control knee pain in the nociceptive pathway [9]. Unfortunately, it remains unclear how many mediators detected in the experiments may activate and/or sensitize human joint nociceptors [9]. The current study suggests that it is not enough for pain-pathology researchers of KOA to involve an isolated phenomenon such as CS; they should consider the complex interactions among polymodal nociceptive sensory neurons and various mediators.

Notably, the subjects in this study demonstrated normal FBG levels on average (non-CS group: 97.1 ± 13.1 mg/dL, CS group: 95.7 ± 15.4 mg/dL). As addressed in the previous paragraph, if we were to evaluate patients with more severe diabetes, DM could be an independent factor associated with deteriorating knee symptoms in patients with radiographic KOA. On the contrary, from the current data, CS and hyperglycemia may synergistically amplify knee pain in radiographic KOA within normal FBG levels [28]. Even if DM is not severe, healthcare providers should pay attention to patients with KOA and comorbid CS. In such cases, controlling FBG can mitigate knee symptoms.

This study has several limitations. First, the mean FBG levels were within the normal range. The Iwaki Health Promotion Project was a health checkup cohort. The participants were relatively wholesome and motivated to keep their body healthier. Therefore, there was a selection bias regarding the patients with diabetes that participated in this study. Future studies to recruit patients with more severe diabetes would clarify the detailed mechanism underlying how hyperglycemia affects knee pain in patients with KOA. Second, the current cohort comprised Japanese Mongoloids. Accordingly, the current results may not be generalizable to other races, such as Caucasians or Negroids. Third, CS was evaluated using only the CSI-9 questionnaire. Quantitative sensory testing (QST) is another way to assess the excitability of pain transduction, transmission, and perception under pathophysiological conditions, such as CS. For instance, pressure pain thresholds [29], temporal summation [30, 31], and conditioned pain modulation [32, 33] are frequently used to assess CS. However, this study did not include QSTs. Despite these limitations, this study clarified the unique pain mechanism by which the combination of hyperglycemia and CS worsens knee symptoms in a population with radiographic KOA. Controlling hyperglycemia has the potential to mitigate knee pain in patients with KOA patients and comorbid CS. Future studies should investigate the detailed mechanisms underlying hyperglycemia and CS in patients with KOA.

## Conclusion

In a Japanese cohort with radiographic KOA, the current study elucidated the relationship between FBG at BL and the longitudinal change in knee symptoms during a one-year follow-up. Higher FBG levels at BL worsened knee symptoms in the patients with radiographic KOA and comorbid CS during 1-year follow-up. In contrast, FBG levels were not longitudinally associated with knee symptoms in patients with radiographic KOA without CS. For subjects with radiographic KOA, the CS comorbidity was negatively associated with KOOS scores at BL. However, CS itself did not affect the longitudinal changes in KOOS. Therefore, comorbid CS worsened knee symptoms in the population with radiographic KOA only when their FBG was elevated.

## Supplementary Information

**Additional file 1: Supplemental Table 1.** Comparison of demographic data between excluded non-OA participants and the current subjects. The values are presented by mean ± SD. Statistical analysis: Chi-square test and Mann–Whitney U-test. (^*^ *P*≤0.05).

## Data Availability

The current database is available only for those who request to testify the validity of this study and acquire the consent from the corresponding author.

## Abbreviations

ADL: Activities of daily living
AGEs: Advanced glycation end products
BL: Baseline
BMI: Body mass index
CS: Central sensitization
CSI-9: Central Sensitization Inventory-9
DM: Diabetes mellitus
FBG: Fasting blood glucose
KLG: Kellgren–Lawrence grade
KOA: Knee osteoarthritis
KOOS: Knee injury and Osteoarthritis Outcome Score
MGO: Methylglyoxal
1YFU: 1-year follow-up
QOL: Quality of life
QST: Quantitative sensory testing
