# Supplementary material for: Higher fasting blood glucose worsens knee symptoms in patients with radiographic knee osteoarthritis and comorbid central sensitization: an Iwaki cohort study
Source: Arthritis Res Ther. 2022 Dec 13;24:269. doi: 10.1186/s13075-022-02951-2 (PMC9745982; doi:10.1186/s13075-022-02951-2)
Supplement: Supplementary file 1 — Additional file 1: Supplemental Table 1. Comparison of demographic data between excluded non-OA participants and the current subjects. The values are presented by mean ± SD. Statistical analysis: Chi-square test and Mann–Whitney U-test. (* P≤0.05). [file 13075_2022_2951_MOESM1_ESM.pptx]

## Slide 1
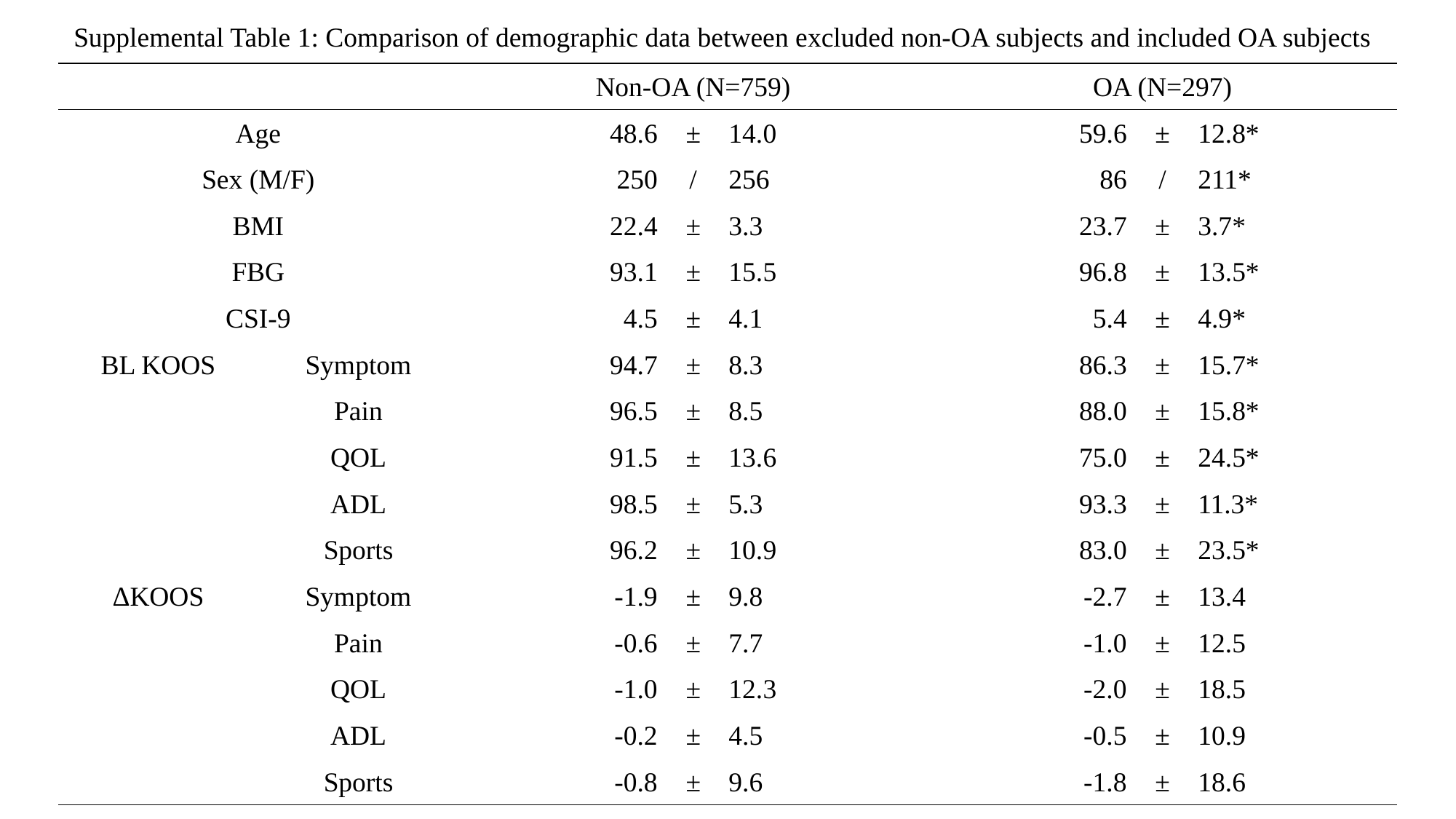

Supplemental Table 1: Comparison of demographic data between excluded non-OA subjects and included OA subjects
| | | Non-OA (N=759) | | | OA (N=297) | | |
| --- | --- | --- | --- | --- | --- | --- | --- |
| Age | | 48.6 | ± | 14.0 | 59.6 | ± | 12.8\* |
| Sex (M/F) | | 250 | / | 256 | 86 | / | 211\* |
| BMI | | 22.4 | ± | 3.3 | 23.7 | ± | 3.7\* |
| FBG | | 93.1 | ± | 15.5 | 96.8 | ± | 13.5\* |
| CSI-9 | | 4.5 | ± | 4.1 | 5.4 | ± | 4.9\* |
| BL KOOS | Symptom | 94.7 | ± | 8.3 | 86.3 | ± | 15.7\* |
| | Pain | 96.5 | ± | 8.5 | 88.0 | ± | 15.8\* |
| | QOL | 91.5 | ± | 13.6 | 75.0 | ± | 24.5\* |
| | ADL | 98.5 | ± | 5.3 | 93.3 | ± | 11.3\* |
| | Sports | 96.2 | ± | 10.9 | 83.0 | ± | 23.5\* |
| ΔKOOS | Symptom | -1.9 | ± | 9.8 | -2.7 | ± | 13.4 |
| | Pain | -0.6 | ± | 7.7 | -1.0 | ± | 12.5 |
| | QOL | -1.0 | ± | 12.3 | -2.0 | ± | 18.5 |
| | ADL | -0.2 | ± | 4.5 | -0.5 | ± | 10.9 |
| | Sports | -0.8 | ± | 9.6 | -1.8 | ± | 18.6 |
